# Maschinelle Autotransfusion in der Geburtshilfe – Hintergrund und praktische Umsetzung

**DOI:** 10.1007/s00101-024-01479-1

**Published:** 2024-11-14

**Authors:** Mischa J. Kotlyar, Vanessa Neef, Florian Rumpf, Patrick Meybohm, Kai Zacharowski, Peter Kranke

**Affiliations:** 1 Klinik und Poliklinik für Anästhesiologie, Intensivmedizin, Notfallmedizin und Schmerztherapie, Universitätsklinikum Würzburg, Oberdürrbacher Str. 6, 97080 Würzburg, Deutschland; 2 Klinik für Anästhesiologie, Intensivmedizin und Schmerztherapie, Goethe-Universität Frankfurt, Universitätsklinikum, Frankfurt, Deutschland

**Keywords:** Kaiserschnitt, Alloimmunisierung, Fruchtwasserembolie, Postpartale Hämorrhagie, Sectio caesarea, Cell saver, Patient blood management, Amniotic fluid embolism, Postpartum hemorrhage, Cesarean section

## Abstract

Die Inzidenz der postpartalen Hämorrhagie (PPH), als eine der führenden Ursachen für die maternale Mortalität, hat in den letzten Jahrzehnten in westlichen Ländern zugenommen. Angesichts der zunehmenden Knappheit und Risiken von Erythrozytenkonzentrattransfusionen bedarf es der Weiterentwicklung fremdblutsparender Maßnahmen in der Geburtshilfe.

Die maschinelle Autotransfusion (MAT), als integraler Bestandteil des Patient Blood Management (PBM), ermöglicht eine Sammlung, Aufbereitung und Retransfusion des patienteneigenen Wundblutes. Trotz zunehmender Evidenz der letzten Jahre, die die Vorteile der MAT aufzeigt, wird das Verfahren bislang bei lediglich 0,07 % aller Geburten mit peripartalen Hämorrhagien an deutschen Krankenhäusern eingesetzt. Es ist anzunehmen, dass der fehlende Einsatz der MAT einerseits auf Sorgen vor patientenbezogenen Risiken wie iatrogene Fruchtwasserembolie und der maternalen Alloimmunisierung während des Einsatzes beruht. Andererseits könnten aber auch die fehlende Anwendung und somit unzureichende Erfahrung im Umgang mit der MAT eine Hürde in deren Nutzung darstellen.

Der nachfolgende Artikel vermittelt einen Überblick über die aktuelle Evidenz zum Einsatz und zur Sicherheit der MAT in der Geburtshilfe. Um eine praxisnahe Umsetzung zu erleichtern, wurden grundsätzliche Überlegungen und organisatorische Vorkehrungen anhand von Erfahrungswerten von den Universitätskliniken Würzburg und Frankfurt übersichtlich aufbereitet und in Form von Grafiken und Checklisten für die perioperative MAT-Anwendung bei Sectio caesarea bereitgestellt.

## Abstract (en)

Postpartum hemorrhage (PPH) remains a leading cause of maternal mortality worldwide, with an increasing incidence in western countries over the past decades. During this period the numbers of PPH-related allogeneic red blood cell (RBC) transfusions have also significantly increased. Given the increasing scarcity and risks of allogeneic RBC transfusions, which are also associated with adverse maternal outcomes, optimized blood management strategies are urgently needed in obstetrics. In recent years, patient blood management (PBM) has been increasingly integrated into medical care, resulting in a significant improvement in patient outcomes. Cell salvage (CS) is one of the PBM blood-sparing techniques that enables the collection, processing and retransfusion of the patientʼs own blood during major bleeding events. Although recent evidence indicates that CS can significantly reduce the demand for allogeneic RBC transfusions and improve patient outcomes, the utilization in obstetrics in German hospitals remains low, with a usage rate of only 0.07% of births with peripartum hemorrhage. It must be assumed that concerns about patient-related complications, such as amniotic fluid embolism and maternal alloimmunization contribute to this hesitancy, alongside a lack of familiarity with the technique. This article provides an overview of the current evidence on the use and safety of CS in obstetrics. To facilitate a practical implementation, fundamental considerations and organizational precautions were prepared based on the experiences of the University Hospitals in Würzburg and Frankfurt and presented in the form of graphics and checklists for the perioperative use of CS during cesarean sections.

## Hinführung zum Thema

Die maschinelle Autotransfusion (MAT), als Teil des Patient Blood Management (PBM), bietet eine effiziente und sichere Möglichkeit, den Fremdblutbedarf während postpartaler Blutungsereignisse zu reduzieren [32, 52, 65, 68]. Trotz zunehmender Evidenz der letzten Jahre, die die Vorteile der MAT aufzeigen, wird das Verfahren u. a. aufgrund von Sorgen vor patientenbezogenen Risiken in der Geburtshilfe an deutschen Krankenhäusern bislang kaum eingesetzt [51].

## Einleitung

Weltweit ist die postpartale Hämorrhagie (PPH) immer noch eine der häufigsten Ursachen für eine erhöhte mütterliche Sterblichkeit [59]. Die PPH-Inzidenz ist in den westlichen Ländern während der letzten Jahrzehnte deutlich gestiegen [4, 20, 33, 34]. Ätiologisch werden u. a. vermehrte Risikoschwangerschaften und gestiegene Sectio-Raten angenommen [33, 40]. Wie beispielsweise Pettersen et al. vom Universitätsklinikum Oslo anhand der Auswertung von 96.313 Geburten berichten, hat an ihrem Standort auch die Transfusionsrate allogener Erythrozytenkonzentrate (EK) im Rahmen postpartaler Hämorrhagien von 12,2/1000 (2008) auf 27,5/1000 (2017) signifikant zugenommen [55]. Zu ähnlichen Ergebnissen kommen auch Studien aus Australien [20] und Kanada [40]. Französische Daten von > 140.000 Geburten (9365 PPH-Fälle), die an 106 Entbindungsstandorten zwischen 2004 und 2006 erhoben wurden, zeigen auf, dass die transfundierte Gesamtmenge mit einem Median von 3 (2–5) EK/PPH-Fall hoch und noch deutlich optimierungsbedürftig ist [10].

In den letzten Jahren hat das Patient Blood Management (PBM) in die medizinische Versorgung Einzug erhalten. Auch in der geburtshilflichen Anästhesie wächst die Bedeutung von PBM [27, 43]. Neben der präpartalen Anämietherapie spielt insbesondere die zweite Säule des PBM, die sich auf fremdblutsparende Maßnahmen beispielsweise mithilfe der maschinellen Autotransfusion (MAT) fokussiert, eine zentrale Rolle bei der Reduktion von Blutverlusten. Die MAT ermöglicht eine Sammlung, Aufbereitung und Retransfusion des patienteneigenen Wundblutes [42].

Gemäß den Guidlines von der Association of Anaesthetists of Great Britain and Ireland (AAGBI) und der European Society of Cardiology (ESC) wird der Einsatz der MAT ab einem potenziell erwarteten Blutverlust ≥ 500 ml empfohlen [23, 30]. Nationale Empfehlungen sehen den MAT-Einsatz als indiziert bei einem „erhöhten Blutungsrisiko“ (Sk2-Leitlinie peripartale Blutungen [2]) bzw. ab einem zu erwartenden Blutverlust >10 % des Körperblutvolumens (Querschnitts-Leitlinie Bundesärztekammer (BÄK) [3]). Dies sei beispielsweise im Rahmen größerer operativer Eingriffe, von Notfalloperationen oder bei entsprechenden Risikofaktoren (Z. n. Uterusatonie oder Plazentaretention, Gerinnungsstörungen etc.) gegeben [2].

Eine kürzlich durchgeführte Observationsstudie an > 300.000 Geburten mit peripartalen Hämorrhagien in einem Kollektiv von über 6 Mio. hospitalisierten Frauen zur Entbindung zeigt, dass die MAT in der Geburtshilfe an deutschen Krankenhäusern bei nur 0,07 % aller Geburten mit peripartaler Blutung und bei 0,31 % aller Kaiserschnittentbindungen mit PPH zum Einsatz kam [51]. Es ist anzunehmen, dass der fehlende Einsatz der MAT in der Geburtshilfe auf Sorgen vor patientenbezogenen Risiken und Komplikationen, wie beispielweise iatrogene Fruchtwasserembolie (FE) oder der Induktion einer maternalen Alloimmunisierung (MAI), während des MAT-Einsatzes beruht. Auch die fehlende Anwendung und somit fehlende Erfahrung im Umgang mit der MAT in der Geburtshilfe können eine erhöhte Hürde in dessen Nutzung darstellen [51].

Der nachfolgende Artikel enthält eine Übersicht über die aktuelle Evidenz zum Einsatz und zur Sicherheit der MAT in der Geburtshilfe. Um eine praxisnahe Umsetzung zu erleichtern, werden grundsätzliche Überlegungen und organisatorische Vorkehrungen für die MAT in der Geburtshilfe übersichtlich aufbereitet sowie eine konkrete Checkliste mit perioperativen Schritten für die MAT-Anwendung bei Sectio caesarea inkludiert.

## Die postpartale Hämorrhagie und der Einsatz von allogenen Bluttransfusionen

Als weltweit führende maternale Todesursache stellt die PPH auch heute noch eine zentrale Herausforderung der Geburtshilfe dar. Ihre Definition variiert. Das American College of Obstetricians and Gynecologists charakterisiert die PPH, unabhängig von der Art der Entbindung, als kumulativen Blutverlust über 1000 ml mit begleitender Symptomatik und objektivierbaren Zeichen einer Hypovolämie innerhalb von 24 h nach Geburt. Die deutsche S2k-Leitlinie peripartale Blutungen stellt einen zusätzlichen Bezug zur Entbindungsart her. Hierzulande gilt die PPH als gegeben, wenn der Blutverlust nach einer vaginalen Entbindung 500 ml übersteigt und nach einem Kaiserschnitt mehr als 1000 ml beträgt [60].

Aufgrund des hohen Blutverlustes geht die PPH mit einer entsprechend hohen und in den letzten Jahrzehnten zunehmenden Rate an EK-Transfusionen (12,2/1000 (2008) auf 27,5/1000 (2017)) als auch einer großen Anzahl transfundierter allogener EK pro PPH-Fall (Median von 3 (2–3)) einher [10, 55]. Wie zahlreiche Untersuchungen zeigen, ist die Transfusion allogener EK mit einem kompromittierten maternalen Outcome assoziiert. Für die Geburtshilfe berichten beispielsweise Kloka et al. auf der Datengrundlage von über 6 Mio. Schwangeren aus Deutschland für den Zeitraum von 2011 bis 2020 von einer signifikanten Häufung von postpartalen Pneumonien (0,5 % vs. < 0,01 %), akutem Nierenversagen (2,1 % vs. 0,4 %) und kardialer Komplikationen (1,2 % vs. 0,4 %) bei Schwangeren, die EK-Transfusionen erhielten, im Vergleich zu nichttransfundierten Kontrollpatientinnen [32]. Auch für venöse Thromboembolien konnte eine dosisabhängige Risikoerhöhung nachgewiesen werden (Odds Ratio: 2,6 (1,7–4,0) für 1–2 EK, 3,6 (1,3–9,6) für mehr als 5 EK) [68].

Angesichts der Assoziation von Transfusionen allogener EK mit einem schlechtem maternalen Outcome und mit Blick auf einen ressourcenschonenden Umgang mit wertvollen allogenen Blutprodukten ist es entscheidend, ihren Einsatz mithilfe von PBM-Strategien auf ein wohlüberlegtes und erforderliches Minimum zu reduzieren. Die MAT kann eine wertvolle Säule im Zuge dieses Bestrebens darstellen.

## Risiken und Nutzen des Einsatzes der MAT in der Geburtshilfe

Das Konzept der maschinellen Autotransfusion ermöglicht die intraoperative Sammlung, Filtration, Waschung sowie die Retransfusion des patienteneignen Blutes. Signifikante Vorteile des MAT-Einsatz konnten beispielsweise durch Meybohm et al. in einer großen Metaanalyse anhand von 47 randomisierten kontrollierten Studien aus dem Jahr 2016 nachgewiesen werden. Dabei konnten mithilfe der MAT in zahlreichen chirurgischen Disziplinen die Exposition der Patienten gegenüber allogenen EK-Transfusion um 39 %, die Rate an postoperativen Infektionen um 28 % sowie die Dauer der Hospitalisierung um 2 Tage reduziert werden [41]. Für spezielle Patientenkollektive mit einer komplexen Antikörperkonstellation, seltener Blutgruppe oder bei Ablehnung von Fremdblutprodukte erhöht das Verfahren die peripartale Sicherheit.

Folgende Risiken müssen beachtet werden.

### Fruchtwasserembolie

Fruchtwasserembolien treten sehr selten auf, gehen allerdings mit einer äußerst hohen maternalen Letalität bis zu 43 % einher [5, 39]. Die pathophysiologische Ursache der FE ist bis heute nicht abschließend geklärt. Historisch postuliert wurde eine pulmonalarterielle Obstruktion durch fetale Fruchtwasserbestandteile; pathognomonisch hierfür sei der Nachweis fetaler Plattenepithelzellen (fPEZ), die in pulmonalarteriellen Gefäßen betroffener Frauen nachgewiesen werden können [14]. Dies führte auch zu einer Verunsicherung in Bezug auf den MAT-Einsatz in der Geburtshilfe, da in deren Blutsammelbehältnis vor und nach dem Waschprozess noch erhöhte Mengen an fPEZ nachgewiesen werden konnten [70], sodass die Induktion einer FE befürchtet wurde. Einerseits zeigt sich jedoch, dass in den einzigen 2 uns bekannten Tierexperimenten an Primaten durch die Injektion auch größerer Mengen an Fruchtwasser und Mekonium eine FE nicht reproduziert werden konnte [1, 62]. Andererseits lassen sich kontroverserweise auch erhöhte Mengen an Plattenepithelzellen in pulmonalarteriellen Gefäßen kritisch kranker Schwangerer mit diversen Erkrankungen nachweisen [15, 16]. In Bezug auf die MAT zeigt sich, dass nach Durchlaufen des Waschprozesses und der Filtration (Leukozytendepletionsfilter, LDF) die Konzentration an fPEZ sowie Bakterien in etwa der im venösen maternalen Blut entspricht [70]. In Zusammenschau erscheint eine mechanische Obstruktion durch Fruchtwasserbestandteile als Ursache einer FE unwahrscheinlich und der Nachweis von fPEZ für eine FE nicht pathognomonisch. Neuere Konzepte zur Entstehung der FE diskutieren immunologische Faktoren, durch vasoaktive und prokoagulatorische Substanzen, die eine inflammatorische Reaktion und Endothelaktivierung auslösen können, sowie die Theorie einer möglichen Aktivierung des Komplementsystems [6, 7, 13]. Aus dem klinischen Blickwinkel liegen zum MAT-Einsatz in der Geburtshilfe Untersuchungen vor, die mehrere Tausend Gebärende umfassen, wobei kein einziger Fall einer FE festgestellt wurde [25, 29, 36, 37, 65]. Erschwerend im Hinblick auf eine schlüssige Kausalpathologie kommt hinzu, dass die Diagnose einer FE bei (über-)lebenden Schwangeren extrem herausfordernd ist und nicht mit absoluter Sicherheit gestellt werden kann.

### Maternale Alloimmunisierung

Während der MAI wird das Immunsystem durch Kontakt zu fetalen Zellen sensibilisiert, sodass Antikörper gegen Antigene fetaler Blutbestandteile produziert werden. Klinisch kann dies eine Alloimmunthrombozytopenie und alloimmunhämolytische Anämie mit entsprechenden Folgen für Feten und Neugeborene nach sich ziehen. Während der MAT können maternale Zellen nicht adäquat von fetalen separiert werden, sodass diese gemeinsam retransfundiert werden. Interessanterweise zeigt sich allerdings, dass hierdurch die Menge an fetalen Zellen im mütterlichen Blutkreislauf im Vergleich zu einer Spontangeburt nicht zunimmt. Die z. T. vorgeschlagene Verwendung zweier Sauger, um während der Wundblutsammlung die erste Fraktion nach der Uterotomie mit Fruchtwasserbestandteilen zu separieren, reduziert nach dem Waschvorgang weder die Anzahl fetaler Zellbestandteilen im Endprodukt signifikant, noch wird das Risiko für die Patientinnen gesenkt. Hingegen resultiert die ausschließliche Verwendung eines einzigen Saugers bzw. Reservoirs („single suction“) in einem ebenso qualitativ hochwertigen und sicheren Blutprodukt, einem größeren retransfundierten Volumen sowie einem niedrigeren logistischen und finanziellen Aufwand [12, 35, 63].

Das Risiko für eine MAT-assoziierte MAI ist mit 1:436 (0,2 %) gering [35] und liegt unterhalb der weltweiten Gesamtprävalenz (0,4–2,7 %) [26, 53, 57]. Wie die AMIGO(Antigen Matching Influence on Gestational Outcomes)-Studie zeigt, werden in 83 % der Fälle vorangegangene Schwangerschaften und in 3 % der Fälle Bluttransfusionen als Ursache mit der MAI assoziiert [18]. Auch wenn demnach eine theoretische Möglichkeit für eine Alloimmunisierung während der MAT besteht, scheint das Risiko hierfür verhältnismäßig gering zu sein, sodass die Vorteile deutlich überwiegen. Unbenommen davon bleibt die Empfehlung eine Standard-Rhesus-Prophylaxe durchzuführen, die ohnehin zum empfohlenen Prozedere nach Sectio rhesusnegativer Mütter mit rhesuspositiven Kindern gehört [11, 22, 67].

### Leukozytendepletionsfilter: mehr Aufwand als Nutzen?

Leukozytendepletionsfilter (LDF) können während einer MAT die Last an Fruchtwasserbestandteilen signifikant reduzieren [58, 70]. Aufgrund mangelnder Evidenz für eine hieraus resultierende Risikoreduktion [29, 35], allerdings wiederholt dokumentierten LDF-assoziierten Komplikationen im Sinne schwerer Hypotonien und Tachykardien [28, 29], für die pathophysiologisch eine Bradykininfreisetzung infolge eines Thrombozytenkontakts mit negativ geladenen LDF diskutiert wird [17, 24], erscheint die Anwendung von LDF nicht sinnvoll, zumal die Nutzung von LDF in einer möglichen Retransfusionsverzögerung bei Transfusionserfordernis größerer Mengen prozessierten Eigenblutes resultieren kann. Auch die S2k-Leitlinie der Deutsche Gesellschaft für Gynäkologie und Geburtshilfe (DGGG) deklariert den LDF-Einsatz als nicht notwendig [60].

## Einsatz und Effizienz der MAT in der Geburtshilfe

In den nationalen und internationalen Leitlinien wurden folgende Indikationen für den MAT-Einsatz definiert: erwartet hohe Blutungswahrscheinlichkeit, präoperative Anämie, unerwarteter intraoperativer Blutungszwischenfall [30, 60]. Das Network for the Advancement of Patient Blood Management, Haemostasis and Thrombosis (NATA) empfiehlt in seiner interdisziplinären Konsensuserklärung, MAT-Systeme in allen größeren Geburtszentren vorzuhalten, sodass diese in relevanten Blutungssituationen, bei erhöhtem PPH-Risiko, Unverfügbarkeit an Blutprodukten aufgrund schwieriger Antikörperkonstellation bzw. seltener Blutgruppe oder der Ablehnung einer Fremdbluttransfusion zum Einsatz kommen können [48].

Die Evidenz für einen sicheren und vorteilhaften MAT-Einsatz bei Schnittentbindungen mit erhöhtem Blutungsrisiko wurde in den letzten Jahren zunehmend gefestigt [19, 37, 52]. Hervorzuheben ist die 2022 veröffentlichte Metaanalyse von Obore et al., für die Daten von 24 Studien und knapp 6000 Frauen mit erhöhtem Blutungsrisiko, die sich einer Sectio unterzogen, ausgewertet wurden. Der MAT-Einsatz war mit einem erhöhten postoperativen Hb sowie einer verkürzten Verweildauer im Krankenhaus assoziiert, während allogene EK-Gaben mit einem 2fach erhöhten relativen Risiko für transfusionsbedingte Nebenwirkungen verbunden waren [52].

Der routinemäßige Einsatz einer MAT bei Schnittentbindungen, unabhängig vom individuellen Blutungsrisiko, wird bislang in nationalen und internationalen Leitlinien nicht empfohlen [30, 60]. Diese Empfehlungen stützen sich dabei v. a. auf Ergebnisse der 2018 veröffentlichten Studie „Cell *Salv*age in *O*bstetrics“ (SALVO), welche keine signifikante Reduktion transfundierter EK durch routinemäßigen MAT-Einsatz nachweisen konnte [29]. Demgegenüber steht allerdings eine in den letzten Jahren zunehmende Fallzahl an Studien, die u. a. eine signifikante Reduktion von Anämiefällen sowie allogener EK-Transfusionen durch eine routinemäßige MAT-Nutzung bei nach Möglichkeit allen Schnittentbindungen aufzeigen konnten [21, 35, 65, 69]. In der Auswertung von Sullivan et al. reduzierte der Einsatz der MAT in einem Zeitraum von 2008 bis 2017 allogene EK-Transfusionen bei Sectiones um mehr als das Dreifache (von 1,4 % auf 0,4 %), während die Retransfusion von MAT-Blut im gleichen Zeitraum von 2,6 % auf 27,2 % zunahm. Am Ende des Beobachtungszeitraumes wurde die MAT bei > 98 % aller Schnittentbindungen eingesetzt [65]. Angesichts dessen, dass die postpartale Anämie in entwickelten Ländern bis zu 50 % der Frauen betrifft [45] und mit einer Erhöhung der maternalen Morbidität und Einschränkung der Lebensqualität aussoziiert wird [45, 50], erscheint es sinnvoll, im Rahmen eines PBM-Konzepts intraoperativ routinemäßig präventive Maßnahmen zu ergreifen, zumal bereits ein Blutverlust > 300 ml mit einer deutlichen Depletion der Eisenspeicher einhergehen und ohne entsprechende Therapie in einer langwierigen Eisenmangelanämie resultieren kann [44]. Diesbezüglich konnten beispielsweise Fox et al. zeigen, dass der routinemäßige MAT-Einsatz bei Kaiserschnitten mit einem höheren postoperativen Hämoglobinspiegel und weniger Anämiefällen assoziiert ist [21].

Bei Sectiones von Schwangeren mit Risikofaktoren für eine PPH traten die positiven Effekte einer konsequenten MAT-Nutzung in der Studie von Wang et al. umso stärker hervor. Die EK-Transfusion-Raten (71 % vs. 25 %) waren ebenso wie das transfundierte Volumen allogenen Blutes (443 ± 386 ml vs. 257 ± 510 ml) in der MAT-Gruppe im Vergleich zur Kontrolle signifikant geringer [69]. Allerdings sollte angemerkt werden, dass unserem Kenntnisstand nach zurzeit kein ausreichend validiertes und für den generellen klinischen Einsatz geeignetes prädiktives Modell für eine PPH existiert [49, 60], wodurch eine zuverlässige Risikostratifizierung bislang nicht möglich ist. Ferner zeigen mehrere Arbeiten, dass der potenzielle Blutverlust bei Kaiserschnitten nicht selten 500 ml und darüber betragen kann [8, 9, 54], sodass entsprechend den Kriterien der AAGBI und der ESC-Guideline der MAT-Einsatz unabhängig vom PPH-Risiko gerechtfertigt sein dürfte [23, 30].

Hinsichtlich des Einflusses einer MAT-Blut-Retransfusion auf die Gerinnungsfunktion bei Kaiserschnittentbindungen deuten mehrere Arbeiten darauf hin, dass hierdurch keine signifikant unterschiedlichen Änderungen unter ähnlichem Bedarf an Fibrinogen, Fresh Frozen Plasma und Thrombozytenkonzentraten zwischen MAT-Kohorten und Vergleichsgruppen hervorgerufen werden [37, 69, 72]. Der MAT-Einsatz ist allerdings mit einem signifikant geringeren Bedarf an allogenen EK assoziiert gewesen [37, 69, 72]. In der prospektiven randomisierten kontrollierten Studie von Liu et al. [37] traten in der MAT-Kohorte ferner Wundheilungsstörungen, allergische Reaktionen, kardiale Ereignisse sowie Hypoproteinämie signifikant seltener auf. Die MAT-Patientinnen waren zudem kürzer hospitalisiert. Aktuell werden international neue Filtersysteme untersucht, die zusätzlich zu Erythrozyten auch Thrombozyten für eine Retransfusion erhalten und das Risiko einer Alloimmunisierung weiter reduzieren sollen [38, 61]. Die Studienergebnisse bleiben abzuwarten.

Das Risiko einer Alloimmunisierung durch eine autologe Retransfusion wird nach dem heutigen Kenntnisstand als gering eingeschätzt, kann allerdings nicht ausgeschlossen werden [35]. Am häufigsten wird eine Alloimmunisierung mit regulären vorausgegangenen Schwangerschaften und allogenen Transfusionen assoziiert [18]. Aus unserer Sicht überwiegen die Vorteile einer Risikoreduktion durch geringere Raten allogener Transfusionen und postpartaler Anämiefälle das geringe Risiko einer potenziellen Alloimmunisierung durch eine MAT. Mit Hinblick auf einen niederschwelligen oder sogar routinemäßigen MAT-Einsatz sollte im Falle einer Rhesusnegativität eine Standard-Rhesus-Prophylaxe ergriffen werden. Da die Exposition gegenüber fetalen Blutzellen durch eine MAT vergleichbar mit der während der peripartalen Phase einer regulären Schwangerschaft zu sein scheint [64], ist aus unserer Sicht eine zusätzliche Dosiskalkulation nicht notwendig. Im Idealfall sollte den betroffenen Frauen ein Follow-up mit Screening auf (irreguläre) Antikörper nach 4 bis 6 Monaten angeboten werden.

Die Autoren der SALVO-Studie schlussfolgerten, dass der routinemäßige MAT-Einsatz mit Hinblick auf die Vermeidung von Fremdbluttransfusionen mutmaßlich nicht kosteneffizient ist [29]. Aus unserer Sicht lassen sich die Kosten im Vergleich zur SALVO-Studie reduzieren, indem u. a. kein Personal ausschließlich für die MAT-Bedienung eingesetzt wird und nur ein Sauger statt 2 genutzt wird, so wie es 58 % der Zentren in der Studie handhabten [29]. Da der MAT-Einsatz ohnehin zunächst mit dem Sammeln des Blutes startet, kann die Eröffnung der Materialien für den Aufbereitungsschritt in Zusammenschau von Blutverlust, gesammeltem Blutvolumen sowie Patientenzustand erfolgen.

In Zusammenschau festigt sich die Evidenz für einen niederschwelligen, wenn nicht gar routinemäßigen MAT-Einsatz bei Schnittentbindungen, um den intraoperativen Blutverlust mit konsekutiver Erhöhung maternaler Morbidität und Einschränkung der Lebensqualität zu minimieren und um den Einsatz von Fremdbluttransfusionen zu reduzieren. Dies könnte sich mittel- bis langfristig auf die bereits knappen Fremdblutressourcen schonend auswirken. Entscheidend zur Erzielung eines Benefits im Sinne einer besseren Rekonvaleszenz ist es – anders als bei der Transfusion von allogenen EK –, einen vergleichsweise liberalen Retransfusionstrigger zu nutzen. Eine sinnvolle Aufbereitung des gesammelten Wundblutes ist aus technischen Gründen ohnehin i. d. R. erst ab einem Volumen ≥ 500 ml möglich. Ferner liegt auch bei Zugrundelegung der sehr restriktiven WHO-Grenzwerte eine Anämie ab einem Hb-Wert unter 11 g/dl vor [71]. Dieser Grenzwert wird u. a. als Indikation für eine Eisensubstitution in der Geburtshilfe herangezogen [46, 47]. Folglich scheint es probat, diesen „Trigger“ als Anhaltswert im perioperativen Kontext zu verwenden, wenn nicht hausintern die Rückgabe jedes noch so kleinen aufbereiteten Blutvolumens festgelegt wird. Letzteres sollte, aus nachvollziehbaren Gründen (u. a. Risiko für Nachblutungen), das Vorgehen darstellen für Patientinnen, die keine Fremdblutgaben erhalten können (Antikörperkonstellation) oder wollen (Zeugen Jehovas). Dieses Vorgehen unterstützt auch die erforderliche Durchdringungsrate in der Übung mit den Devices seitens des involvierten Personals. Wenn die MAT nicht in anderen Bereichen (z. B. Gefäßchirurgie, Leberchirurgie etc.) entsprechend frequent eingesetzt wird oder die Anästhesie- bzw. OP-Teams nicht universell eingesetzt werden, erscheint es ratsam, die gewünschte Anwendervertrautheit durch eine Routine-Anwendung zu erzielen.

Am Rande sei erwähnt, dass die MAT auch während vaginalen Entbindungen sicher und effizient eingesetzt werden kann [56, 66].

## Praktische Umsetzung

**Abb. 1:**
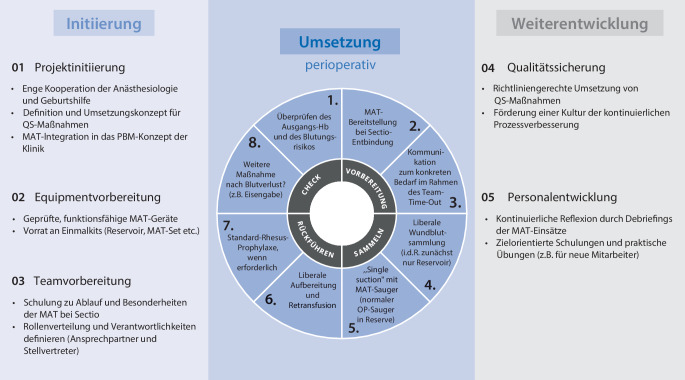
Übersicht über aus Sicht der Autoren notwendige Schritte vor einer ersten MAT-Anwendung, das perioperative Vorgehen und Möglichkeiten der Projektweiterentwicklung. *MAT* maschinelle Autotransfusion, *PBM* Patient Blood Management, *QS-Maßnahmen* Qualitätssicherungsmaßnahmen

Die MAT ist ein Verfahren, bei dem das während einer Operation verlorene Blut der Patientin gesammelt, aufbereitet und retransfundiert wird. Dies kann insbesondere bei einer Sectio caesarea von Vorteil sein, um die Wahrscheinlichkeiten für eine peripartale Anämie und Fremdbluttransfusionen zu minimieren. Die Implementierung der MAT bei einer Sectio caesarea erfordert sorgfältige Planung und Durchführung, um Sicherheit und Effektivität zu gewährleisten. Eine Übersicht über die aus unserer Sicht notwendigen Schritte vor einer ersten Anwendung und ein mögliches Vorgehen zur Projektweiterentwicklung kann Abb. 1 entnommen werden.

Ferner fassen Abb. 1 und 2 die wichtigsten Punkte zum perioperativen Vorgehen bei einer Sectio caesarea zusammen.

**Abb. 2:**
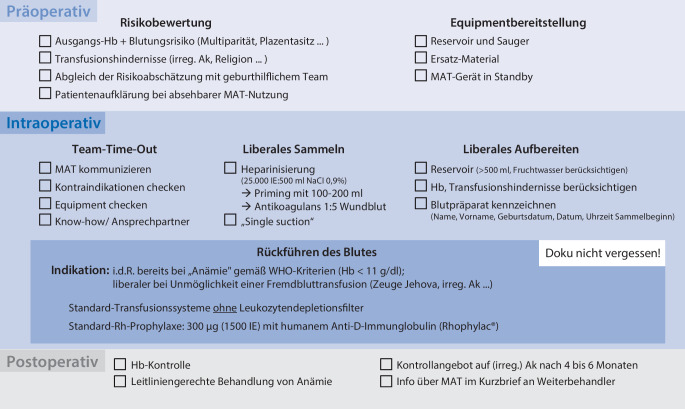
Checkliste für aus Sicht der Autoren relevanteste perioperative Schritte rund um die MAT-Anwendung bei einer Sectio caesarea. *Ak* Antikörper, *EK* Erythrozytenkonzentrate, *Hb* Hämoglobin, *MAT* maschinelle Autotransfusion, *Rh* Rhesus

## MAT-Projekt: Initiierung, Umsetzung und Weiterentwicklung

Die *Initiierungsphase* sollte aus unserer Sicht durch eine enge Zusammenarbeit zwischen den verantwortlichen Abteilungen der Geburtshilfe und Anästhesiologie geprägt sein. Vorteilhaft ist die Erfahrung mit MAT in anderen chirurgischen Disziplinen. Es ist wichtig, bereits zu Beginn klare Strukturen zu schaffen, Verantwortlichkeiten zu definieren und das MAT-Projekt in das PBM-Konzept des Krankenhauses zu integrieren. Auch wenn es mitunter als Hürde empfunden wird, ist es unabdingbar, ein Umsetzungskonzept für die vorgeschriebenen Qualitätssicherungsmaßnahmen konsequent festzulegen. Bevor der erste Einsatz erfolgen kann, muss sichergestellt werden, dass funktionsfähige MAT-Geräte sowie Einmalkits mit Reservoir, Sets etc. in ausreichender Menge bevorratet werden. Eine Teamschulung hinsichtlich der Besonderheiten während eines MAT-Einsatzes bei Sectiones ist von großer Bedeutung. Da im Verlauf der Integrationsphase der MAT in den klinischen Alltag häufig anwendungsbezogene Fragen aufkommen können, sollten hierfür ein fester Ansprechpartner sowie dessen Vertreter festgelegt werden.

Die *Umsetzungsphase *lässt sich grob in 3 Abschnitte gliedern – präoperativ, intraoperativ sowie postoperativ.

*Die präoperative Phase* ist insbesondere durch eine konsequente Vorbereitung des Equipments und der interdisziplinären Risikobeurteilung hinsichtlich des Blutungsrisikos (Multiparität, Plazentasitz etc.) und der Transfusionshindernisse (irreguläre Antikörper, Religion) der Patientin geprägt. Bei absehbarer MAT-Nutzung sollte die Patientin hierüber aufgeklärt werden.

*Intraoperativ* ist nochmals drauf zu achten, dass während des Team-Time-Out das gesamte Operationsteam informiert wird und das notwendige Equipment funktionsfähig bereitsteht. Bevor der Sammelvorgang beginnt, müssen Schläuche und das Reservoir mit 100–200 ml einer heparinhaltigen Infusionslösung (25.000 IE auf 500 ml 0,9 %ige NaCl-Lösung) benetzt werden, um einer Koagulation des Blutes vorzubeugen. Anschließend empfiehlt es sich, während des Sammelvorgangs mit einem Mischverhältnis von ca. 1:5 zwischen Antikoagulans und Wundblut fortzufahren. Aus unserer Sicht sollte, wie bereits zuvor erläutert, für den Sammelvorgang ein Einsaugersystem („single suction“) genutzt werden, während ein Standard-OP-Sauger als Back-up bereitsteht, z. B. im Falle eines Clotting des MAT-Saugers; dies entspannt die Situation ungemein. Ebenso wie das Sammeln sollten auch das Aufbereiten und die Retransfusion liberal erfolgen, insbesondere wenn bereits präoperativ ein niedriger Hb vorlag und zwingend dann, wenn Transfusionshindernisse bestehen. Eine Aufbereitung ist ab 500 ml Wundblut im Reservoir möglich. Während des gesamten MAT-Einsatzes sollte auf eine entsprechende Dokumentation geachtet werden. Das Blutpräparat muss konsequent mit Patientendaten gekennzeichnet werden. Bevor eine Retransfusion des autologen EK erfolgt, sollte patientenseitig eine BGA zur Anämiedetektion abgenommen werden. Die Retransfusion sollte aus unserer Sicht aufgrund mangelnder Evidenz zu Vorteilen, allerdings erhöhtem logistischen Aufwand und möglicher Zeitverzögerung ohne einen Leukozytendepletionsfilter erfolgen. Die Rhesusprophylaxe erfolgt standardmäßig mit 300 µg (1500 IE) humanem Anti-D-Immunglobulin (Rhophylac®). Von dem Versuch einer quantitativen Bestimmung des Anteils fetaler Erythrozyten im mütterlichen Blut, z. B. mittels Kleihauer-Betke-Tests [31], sind wir mangels Handlungskonsequenz abgekommen.

*Postoperativ *ist eine erneute Hb-Kontrolle indiziert. Bei Detektion eines Substratmangels (Eisenmangel, Vitamin B_12_, Folsäure) in der Anämiediagnostik sollte eine entsprechende Substitution eingeleitet werden, um die Hämatopoese zu unterstützen. Der MAT-Einsatz ist für Weiterbehandler ersichtlich zu dokumentieren. Außerdem ist es aus unserer Sicht sinnvoll, der Patientin einen Kontrolltermin zum Screening nach (irregulären) Antikörpern in 4 bis 6 Monaten anzubieten.

Eine konkrete Checkliste für die prä-, intra- und postoperative Phase ist in Abb. 2 dargestellt. Die Checkliste erhebt keinen Anspruch auf Vollständigkeit, sondern beinhaltet die aus unserer Sicht relevantesten perioperativen Schritte rund um die MAT-Anwendung.

Eine erfolgreiche Initiierungsphase und erste Einsätze sollten optimalerweise von einem *Weiterentwicklungskonzept* abgerundet werden. Hierfür bietet es sich an, neben den vorgegebenen Qualitätssicherungsmaßnahmen Kriterien für eine Erfolgsbeurteilung, ggf. auch eine systematische Datensammlung und -analyse, festzulegen. Aus unserer Sicht ist es ferner vorteilhaft, im persönlichen Gespräch Schwierigkeiten und offene Fragen rund um abgelaufene MAT-Einsätze im Team zu diskutieren und entsprechende Lösungskonzepte zu implementieren.

## Empfehlungen der Bundesärztekammer

Die BÄK beschreibt in ihrer 2023 veröffentlichten Richtlinie für Hämotherapie u. a. die wichtigsten Verfahrensrichtlinien für die Qualitätssicherung der MAT [11]. Grundsätzlich sollte laut BÄK der MAT-Einsatz immer dann erwogen werden, wenn ein hoher Blutverlust zu erwarten ist.

Relevanter Grundsatz ist, dass durch die MAT kein lagerungsfähiges Produkt entsteht. Dementsprechend muss eine Retransfusion spätestens 6 h nach dem Sammelbeginn erfolgen. Die hergestellten Blutpräparate sind mit Namen, Vornamen und Geburtsdatum des Patienten sowie Datum und Uhrzeit des Beginns der Sammlung zu kennzeichnen. Für jede maschinelle Autotransfusion müssen zudem folgende Informationen dokumentiert werden: Patientendaten, verantwortlicher Arzt, Beginn und Ende der Prozedur, Typ und Nummer des verwendeten Geräts, Chargennummern verwendeter Aufbereitungssysteme, Volumen des gesammelten Blutes und des aufbereiteten Blutes.

Jeder Anwender ist in die MAT und die Aufbereitung von Wundblut einzuweisen. Vor jeder MAT-Anwendung sind entsprechende absolute und relative Kontraindikationen sowie Notwendigkeiten zur spezifischen Aufbereitung zu beachten.

Bei Nachweis einer bakteriellen Kontamination des Blutes (Infektion im Operationsgebiet, Sepsis) ist die MAT nicht zulässig.

Zur Qualitätssicherung des MAT-Verfahrens sollen bei 5 % aller Einsätze, mindestens einmal pro Monat und Gerät sowohl der Hämatokritwert am aufbereiteten Präparat (Ziel > 50 %) und die Eliminationsrate von Gesamteiweiß oder Albumin (Sollwert > 90 % des Ausgangswertes) bestimmt werden.

## Zusammenfassung

Die Inzidenz postpartaler Hämorrhagien hat trotz steigender medizinischer Standards in den vergangenen Jahrzehnten zugenommen und stellt damit auch heutzutage noch eine zentrale Herausforderung der Geburtshilfe dar. Die PPH ist mit einem hohen maternalen Blutverlust sowie einem entsprechend hohen Transfusionsbedarf allogener Erythrozytenkonzentrate vergesellschaftet. Die maschinelle Autotransfusion bietet als integraler Bestandteil des Patient Blood Management eine evidenzbasierte, effiziente und sichere Möglichkeit, um den Fremdblutbedarf und damit einhergehende Risiken bei erwartet hohem Blutverlust oder Transfusionshindernissen während eines Kaiserschnitts zu reduzieren. Neuere Studienerkenntnisse weisen ferner darauf hin, dass auch der routinemäßige MAT-Einsatz bei Schnittentbindungen vorteilhaft sein kann. Die Implementierung der MAT bei einer Sectio caesarea erfordert eine sorgfältige Planung und Durchführung, um Sicherheit und Effektivität insbesondere im Fall starker Blutungen zu gewährleisten. Um die erforderliche Routine im Kreise der Mitarbeiter aufrechtzuerhalten, sollte das Verfahren regelmäßig eingesetzt und das innerklinische MAT-Konzept fortwährend weiterentwickelt werden.

## Fazit für die Praxis

- Die PPH-Inzidenz hat in westlichen Ländern während der letzten Jahrzehnte ebenso wie die mit ihr verbundene peripartale Transfusionsrate allogener EK signifikant zugenommen.
- Die MAT bietet als integraler Bestandteil des PBM eine evidenzbasierte, effiziente und sichere Möglichkeit, um den Fremdblutbedarf bei erwartet hohem Blutverlust oder Transfusionshindernissen während eines Kaiserschnitts zu reduzieren.
- Die Evidenz für einen niederschwelligen, wenn nicht gar routinemäßigen MAT-Einsatz bei Schnittentbindungen wurde in den letzten Jahren gefestigt.
